# Relationship between Sn elemental background values and regional longevity levels—Data from Yunnan, China

**DOI:** 10.1371/journal.pone.0332369

**Published:** 2025-09-12

**Authors:** Shuangshuang Zhu, Jiaxue Wang, Xin Zhou, Min Lai, Changqing Peng

**Affiliations:** Faculty of Geography, Yunnan Normal University, Kunming, China; Southwest Forestry University, CHINA

## Abstract

The relationship between the geographic environment and human health has been a long-standing focus of scientific inquiry. Sn as an essential trace element for the human body, play vital roles in individual health and may influence longevity. However, the extent to which the statistical characteristics of population longevity are associated with elemental geochemical background values at a regional scale remains an important question. Based on the geochemical survey data of Yunnan Province and Chinese census data, the article utilizes Arcgis spatial analysis and mathematical statistics to explore the relationship between ω(Sn) and regional longevity level. The results of the study show that: (1) There is a close correlation between ω(Sn) and regional longevity levels. Within Yunnan Province, regions with high ω(Sn) have higher levels of longevity index and Ultra-octogenarian Index. (2) Spearman’s correlation coefficient shows that ω(Sn) is significantly positively (P < 0.01) correlated with both the longevity index and the Ultra-octogenarian Index; Linear regression further reveals that ω(Sn) always has a significant positive influence on the longevity index. For the Ultra-octogenarian Index, although the strength of the influence of ω(Sn) is not as significant as that of the longevity index, its influence on the healthy longevity of the population cannot be ignored. At the county scale in Yunnan Province, there is a significant positive correlation between ω(Sn) and longevity index, which may be related to the exposure of Sn in the natural environmental background into the human body and thus affecting the incidence of cancer, but the biogeochemical cycling mechanism of its association with longevity still needs to be further investigated.

## Introduction

Healthy and long life has always been the unremitting pursuit of human beings, as well as the eternal theme and ultimate challenge of life science research. Human longevity is closely related to the environment [1,2], genetics [3–5] and lifestyle, and among these factors, the environment is particularly important [6–9]. In particular, trace elements and minerals in the natural environment can affect the quality of drinking water and food, and have a significant impact on human health and longevity [10,11]. Elements as the basic components of the human body, but the human body is unable to produce trace elements on its own, and its intake must come from the natural environment [12]. Therefore, the content of trace elements in the natural environment has a significant impact on human health and longevity. In recent years, a number of important results have been achieved in related research. For example, Olawoyin et al. assessed the levels of heavy metals in the soil environment of the Niger River region and their potential risk to human health, showing that when Pb and Cr concentrations in the soil environment reach a certain level, they can threaten human health [13]. Lar and Uriah in their study on Nigeria found that the level of environmental concentrations of elements such as Pb, Cu, Zn, As, Cd, and Hg pose potential risks to human health [14]; And a study by Yang et al. found a significant correlation between trace elements in soil (e.g., Se, Zn, B, etc.) and the proportion of long-lived population [15]. In addition, Chen et al [16–18]. showed that these grains are rich in the major elements Ca, Na, Mg, P, and S, as well as the trace elements Cr, Mo, Ca, Mg, and Se, through a study of wheat in the longevity area of Yunnan Province, and their intake is closely related to longevity.

It has also been shown that there are distributional similarities between the proportion of the Chinese population aged 90 years and over and some elements in the soil (e.g., Se and Zn). However, uneven distribution of trace elements in the natural environment may also lead to endemic diseases, such as fluorosis [19], selenium toxicity [20], Kaschin-Beck disease, and Keshan disease [20] and that strict control of elemental concentrations may promote regional health and longevity, such as Zhongxiang County, China [12], and the longevity area of Mengshan Mountain, China [21]. Sn is one of the trace elements necessary for human life activities, it has a close relationship with human health, the normal human body contains about 17 mg of Sn [22]. Generally speaking, on the scale of individual health and longevity and natural geography, the human body’s intake of Sn is mainly derived from food and water, which has an important impact on the human body to carry out physiological activities and maintain human health [23]. Appropriate Sn can enhance the stability of the body’s internal environment and play a key role in promoting human growth and development. However, both Sn deficiency and high Sn can affect human health. When the intake dose is too small, it can cause slow development of human body and even induce dwarfism [24]; when the intake dose is too large, it can cause toxicity symptoms, which are accompanied by headache, memory loss or impairment in severe cases [25]. It has also been shown that Sn has the effect of inhibiting cancer cells [26], such as Kikuchi et al [27] who found a significant correlation between the incidence of colorectal cancer and the concentration of Sn in water.

The above research results provide an important scientific basis in disease prevention and treatment, however, whether there is also a significant correlation between Sn in the natural environment and regional longevity levels at the regional scale is still a scientific proposition worthy of in-depth study. Clarifying the relationship between Sn in the natural environment and the regional longevity level is an important guideline for the formulation of regional public health policies, and it also helps to promote the development of health and wellness tourism products related to Sn, which in turn promotes the synergistic development of the regional health and tourism industries.

Regional longevity levels are influenced by a combination of factors, including geochemical elements in the natural environment, genes, living habits, local culture, medical care, and other natural factors [8,28,29]. In geographically complex areas, these factors are intertwined and have a more pronounced effect on longevity levels. The credibility of the findings of this study will be enhanced if the correlation between Sn in the natural environment and longevity levels can still be observed in such complex environments. Yunnan Province, China, located on the southeastern edge of the Tibetan Plateau, is one of the few regions in the world that is characterized by simultaneous geological, soil, biological, mineral, and ethnocultural diversity. Given this diversity, the selection of Yunnan Province for the study of the relationship between Sn background values (ω(Sn)) and longevity regions helps to demonstrate the real link between ω(Sn) and longevity phenomena even under different soil types, ecological environments, cultures and living conditions.

## Overview of the study area

### Physical and geographic profile

**Fig 1:**
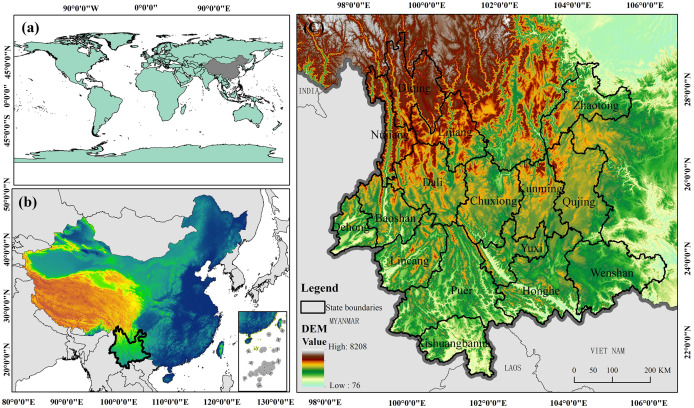
Schematic diagram of the study area. (a: China’s position in Asia; b: Yunnan’s position in China; c: Yunnan Province). Created using ArcGIS 10.2.

Yunnan Province is located in southwestern China, between longitudes 97°31′ and 106°11′ E and latitudes 21°8′ and 29°15′ N (Fig 1), with the Tropic of Cancer crossing the southern part of the province. The maximum horizontal distance from east to west in the province measures 864.9 kilometers, while the maximum vertical distance from north to south spans 990 kilometers. Yunnan covers a total area of approximately 394,100 km^2^, representing 4.1% of China’s total land area, and ranks eighth in terms of area among all provincial-level administrations in China. The region is situated in the collision zone between the Asia-Europe Plate and the Indian Ocean Plate, enduring long-term influences of internal and external forces that have shaped its diverse geological landforms. Owing to its intricate geology and distinctive geographical positioning, the region hosts a broad spectrum of climate types conducive to the survival and proliferation of various organisms. Over time, the area has undergone extensive geological processes involving crustal movements, magmatic activities, and sedimentation, fostering the creation and diversity of mineral resources. Variances in climate, soil composition, vegetation, terrain, and human interventions significantly impact the distribution of soil types and trace elements.

### Socio-economic profile

Yunnan Province is one of the important birthplaces of humankind. By the end of 2023, the resident population of Yunnan Province had reached 46.73 million, with 24.73 million residing in urban areas and 22 million in rural regions, leading to an urbanization rate of 52.92%. Although the region’s economy is primarily agriculture-based, the flourishing tourism sector, non-ferrous metal industry, bio-resource development, and other industries have emerged as vital drivers of economic growth. At the same time, Yunnan Province is a key window for China’s opening to the outside world and maintains close economic and cultural exchanges with neighboring countries. As one of the provinces with the largest number of ethnic minorities in China, Yunnan is home to 26 ethnic groups, which display significant differences in their production and life, social organization, literature and art, and religious ceremonies. The complex topography of Yunnan Province, which is predominantly mountainous, leads to inconvenient transportation between the various states, which in turn affects the frequency of economic exchanges, thus presenting regional economic disparities and uneven distribution of medical resources. There are obvious differences between cultural diversity, production and lifestyle, economic development level and medical care level, providing a rich research sample for this study.

## Data and methods

### Data source and preprocessing

#### Element background value data.

Sn in the natural environment may originate from a variety of media such as soil, rock, atmosphere, organisms, and water environment. Obtaining comprehensive and accurate Sn data in a provincial area of more than 390,000 square kilometers is a challenging task. In view of this, in order to preliminarily investigate the statistical relationship between Sn and population longevity level, the geochemical background value of Sn was selected as the entry point to carry out the relevant study. The Sn background values for each county-level administrative region are derived from the “Geophysical and Geochemical Atlas of Yunnan Province” completed by the Yunnan Provincial Bureau of Geology and Mineral Resources between 1979 and 1999. The results were based on aquatic sediments as the main sampling medium, supplemented only by soil samples in a few missing areas, with a sampling density of 1 ~ 2 samples/km² and a large compartmentalization of samples in 4 km², and a total of 96,399 samples were collected. The determination and analysis of the samples strictly followed the “Provisions on Regional Geochemical Surveys” and the methods uniformly deployed in China, and were compiled into maps accordingly [30]. During the data collection period, the overall socio-economic development level of Yunnan Province was low, pesticides and fertilizers were not yet used on a large scale, and there was less interference from human activities, Therefore, this data can accurately reflect the Sn geochemical background values in various counties in Yunnan Province.

The article is in the process of data processing, the acquired raw images are firstly rasterized and geographically aligned to ensure accurate geospatial positioning of the images. Subsequently, the aligned images are digitized and the corresponding attribute information is recorded. Next, the digitized image is converted to vectorization, and the vectorization results are checked for topology and accuracy of attribute entry. Finally, targeted technical measures are taken to deal with problems that may arise during the operation, such as missing vectorization, boundary mismatch, missing plates, unmerged identical patches, and overlapping elements, etc., in order to ensure the reliability and accuracy of the data. In this study, the average background value of 3.3 mg/kg of sediment in Chinese water system [30] and 5.85 mg/kg of sediment in Yunnan Province water system [31] were used as the reference basis for comparing and analyzing the relevant data.

#### Population data and longevity indicators.

The article’s population data comes from the China National Bureau of Statistics Fifth, Sixth, and Seventh Population Census Database, China Population Census Yearbook [32–34]. The article uses the longevity index [35] (LI‰, the proportion of population over 90 years old to the proportion of population over 65 years old) and the Ultra-octogenarian Index (UOI%, the proportion of population over 80 years old to the proportion of population over 60 years old) to assess the longevity phenomenon in a region. The longevity Index has good accuracy in general, shows good stability and applicability in small-scale regions, and can effectively avoid the problem of indicator fluctuation due to small population size [36]. The Ultra-octogenarian Index can visualize the longevity status of a region or group and help to compare the differences between different regions and time periods. Among the 129 counties in Yunnan Province, there are 104 counties with populations less than 500,000, so this paper mainly selects LI‰ and UOI% to measure the regional longevity level.

### Research methods

#### Global autocorrelation analysis.

Global spatial autocorrelation analysis is a spatial statistical method used to measure whether observations in geospatial data have similarity to those in their neighboring regions. He mainly determines whether the data are randomly distributed in space or whether there is some kind of aggregation or discrete trend [37]. By comparing the mean value of ω(Sn) in the study area with the ω(Sn) in each county, it is concluded whether ω(Sn) is clustered or not in the region, and its formula [38,39] is as shown in Equation 1:


$$I=\frac{n.\sum_{i=1}^{n}\sum_{j=1}^{n}w_{ij}(x_{i}-x)(x_{j}-x)}{(\sum_{i=1}^{n}\sum_{j=1}^{n}w_{ij}).\sum_{i=1}^{n}{\left(x_{i}-x\right)}^{2}},i\neq j$$ (1)

In the formula, I represents the Moran’s I index; n denotes the total number of study units, i.e., the total number of counties; *x*_*i*_ and *x*_*j*_ are the attribute values of the i-th and j-th regions, respectively, representing the ω(Sn) values of the counties; $\bar{\text{x}}$ is the mean of all regional attribute values, i.e., the average ω(Sn) value across all counties; $w_{ij}$ is the spatial weight matrix, which represents the adjacency relationships of the spatial objects. The Moran’s I index ranges between [−1, 1]: I > 0: indicates positive spatial autocorrelation, meaning ω(Sn) exhibits clustering in its spatial distribution. I = 0: indicates a random spatial distribution. I < 0: indicates negative spatial autocorrelation, meaning ω(Sn) is dispersed in its spatial distribution Equation 2.

The standardized test statistic Z is used to evaluate the significance of Moran’s I index, and its formula is as shown in Equation 2:


$$z=\frac{1-E\left(I\right)}{\sqrt{VAR\left(I\right)}}=\frac{\sum {\mathrm{\backslash nolimits}}_{j\neq 1}^{n}W_{ij}\left(d\right)\left(x_{j}-{\bar{x}}_{i}\right)}{\sqrt[{S_{i}}]{\frac{w_{i}\left(n-1-w_{i}\right)}{n-2}}},\left(i\neq j\right)$$ (2)

#### Hotspot analysis.

The Getis-statistic is computed for each county ω(Sn), and the resulting Z-score and P-value indicate where spatially clustering occurs for counties with high or low values of ω(Sn), and its formula [40] is as shown in Equation 3:


$$G_{i}^{*}=\frac{\sum_{j=1}^{n}w_{i,j}x_{j}-x\sum_{j=1}^{n}w_{i,j}}{\sqrt{\frac{\sum_{j=1}^{n}w_{j}^{2}}{n}}-x^{2}\sqrt{\frac{n\sum_{j=1}^{n}w_{i,j}^{2}-({\sum_{j=1}^{n}w_{i,j})}^{2}}{n-1}}}$$ (3)

In the formula, the meaning of the symbols is the same as before. $G_{i}^{*}$ statistics is the Z score, the larger the absolute value, the higher the degree; greater than 0 indicates a positive hot spot, belonging to the ω (Sn) of the larger counties gathered in the region; less than 0 indicates a negative hot spot, belonging to the ω(Sn) small counties gathered in the region; close to 0, then a random distribution.

#### Clustering and outlier analysis.

The spatial clustering pattern of counties with higher or lower ω(Sn) in the study area can be identified by using Local Moran’s I metrics, and its formula [41,42] is as shown in Equation 4:


$$I_{i}=\frac{x_{i}-x}{\frac{\sum_{i=1,j\neq 1}^{n}{\left(x_{j}-x\right)}^{2}}{n-1}x^{2}}\sum_{i=1,j\neq 1}^{n}w_{i,j(x_{j}-x)}$$ (4)

The statistical score is calculated according to Equation 5:


$$Z_{I_{i}}=\frac{I_{i}-E[I_{i}]}{\sqrt{\left(E[{I_{i}}^{2}]-E{\left[I_{i}\right]}^{2}\right)}}$$ (5)

Of which $E[I_{i}]$ is as shown in Equation 6:


$$E\left[I_{i}\right]=-\frac{\sum_{j=1,j\neq i}^{n}w_{ij}}{n-1}$$ (6)

where *X*_*i*_ and *X*_*j*_ denote the observed values of a phenomenon on space units i and j, respectively; the rest of the symbols have the same meaning as before. An element is part of a cluster if a positive value of I indicates that the element has neighboring elements that contain equally high or low values of the attribute; an element is an outlier if a negative value of I indicates that the element has neighboring elements that contain no values.

The spatial distribution of county ω (Sn) in Yunnan Province was analyzed using the clustering and outlier analysis tools of ArcGIS 10.2, and the statistical significance of the index was assessed by Local Moran’s I index values, Z-score、P-value, and the cluster type codes that denote each element of the, And two clustering patterns, high value (HH) clustering and low value (LL) clustering, as well as two types of outliers, low values surrounded by high values (LH) and high values surrounded by low values (HL), were derived.

### Spatial overlay analysis

Spatial overlay analysis, also known as “spatial superposition analysis”, is one of the core techniques in geographic information systems (GIS), which is to superimpose the geometric and attribute information of multiple spatial layers of the same area under a unified spatial reference system to produce multiple attribute characteristics of spatial regions or to establish spatial correspondences between different geographic objects [43], so as to better reveal the spatial correlation law between ω (Sn) and the regional longevity level.

### Spearman rank correlation

Spearman’s rank correlation analysis is a statistical method for measuring the degree of correlation between two variables. The Spearman correlation coefficient is *r*_*s*_ represented by, with values ranging from [-1,1]. The greater the absolute value, the stronger the correlation. *r*_*s*_ = -1 indicates a perfect negative correlation, *r*_*s*_ = 1 indicates a perfect positive correlation, *r*_*s*_ = 0 indicates no linear relationship. The formula [44] is as shown in Equation 7:


$$r_{s}=1-\frac{6\sum {\mathrm{\backslash nolimits}}_{i=1}^{N}d_{i}^{2}}{N\left(N^{2}-1\right)}$$ (7)

In the formula: *r*_*s*_ is the Spearman correlation coefficient, *d*_*i*_ is the rank difference between the i observed values of the two variables (i.e., the rank difference between *X*_*i*_ and *Y*_*i*_, where *X*_*i*_ and *Y*_*i*_ are the observed values of the two variables), and is the sample size.

## Results

### Spatial distribution characteristics of ω(Sn)

**Fig 2:**
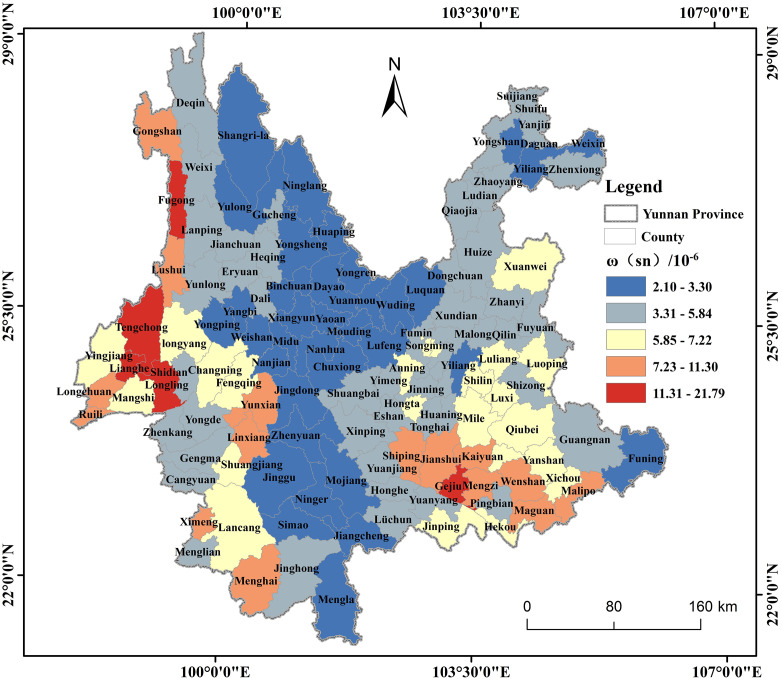
Distribution map of ω (Sn) in counties in Yunnan Province. Created using ArcGIS 10.2.

Using ArcGIS 10.2, the Sn geochemical map of Yunnan Province was converted into raster data. The Zonal Statistics tool applied to calculate the average ω(Sn) values for the 129 counties within Yunnan Province. The sediment background values of 3.3 mg/kg for Chinese water systems and 5.85 mg/kg for Yunnan water systems were utilized as references to define the ranges of Interval 1 (2.10 mg/kg – 3.30 mg/kg) and Interval 2 (3.31 mg/kg – 5.85 mg/kg). Values exceeding 5.85 were categorized using the natural break classification method, resulting in a total of five intervals (Fig 2). Among the 129 counties in Yunnan Province, 96 counties exhibit ω(Sn) values exceeding the background value of 3.3 mg/kg for sediment in Chinese water systems, accounting for 74.4% of the total. These counties are primarily concentrated in the eastern and southwest regions of Yunnan Province. In the eastern region, they include most counties in Qujing City, Yuxi City, Wenshan Zhuang and Miao Autonomous Prefecture, Honghe Hani and Yi Autonomous Prefecture, Kunming City, and a significant portion of counties in Zhaotong. In the southwestern region, they encompass Dehong Dai and Jingpo Autonomous Prefecture, Lincang City, Baoshan City, Deqin Tibetan Autonomous Prefecture’s Deqin County, Weixi Lisu Autonomous County, western Jianchuan County in Dali Bai Autonomous Prefecture, Heqing County, Eryuan County, as well as Menglian County, Lancang County, and Ximeng County in Pu’er City. The highest value recorded is 21.79 mg/kg, observed in Gejiu City of Honghe Hani and Yi Autonomous Prefecture. Among them, 33 counties, or 25.6%, had ω(Sn) lower than the background value of 3.3 mg/kg for sediment in Chinese water systems.It is mainly distributed in most counties of Chuxiong Prefecture, Lijiang City and Pu’er City in central Yunnan Province.as well as Daguan County, Yiliang County, Weixin County in Zhaotong and Funing County in Wenshan Prefecture. The lowest value was 2.10 mg/kg, which occurred in Mengla County, Xishuangbanna Prefecture.

In order to deeply explore the spatial clustering of ω(Sn), a global autocorrelation analysis was conducted at the county scale in Yunnan Province (Table 1). The Moran’s I index of ω(Sn) is 0.256, which is larger than the respective corresponding expected index, the P value is less than 0.01, which is in the confidence space of more than 99%, and the Z score is positive and larger than 1.96, which indicates that there exists an obvious spatial positive correlation of the distribution of ω(Sn) on the county scale, i.e., there exists a clustering characteristic in the spatial distribution, which is not a random distribution in space.

**Table 1:** Global autocorrelation analysis of ω(Sn).

ω(Sn)	Moran’s I	Expectation index	variance	Z score	P level	Result
0.256	−0.007812	0.002355	5.448	<0.001	congregate

### Spatial distribution pattern of ω(Sn)

**Fig 3:**
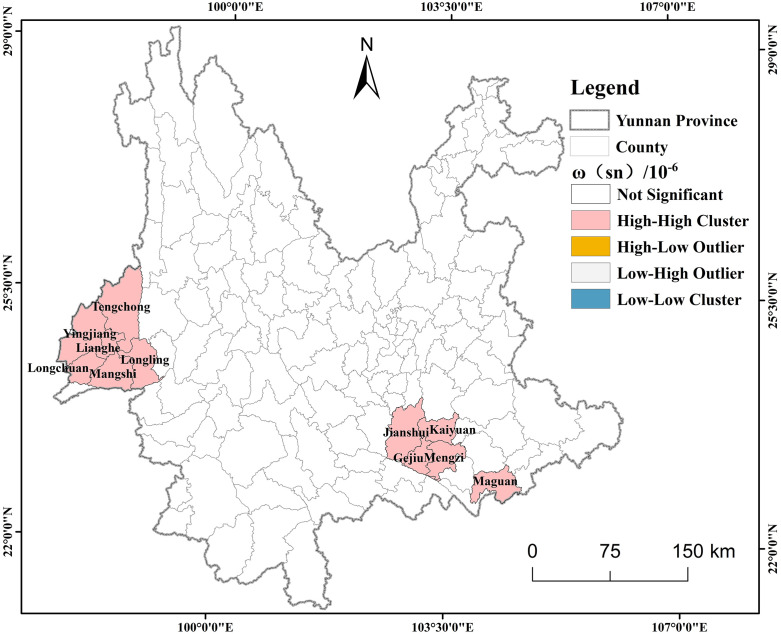
Local spatial autocorrelation maps of ω(Sn) in Yunnan Province. Created using ArcGIS 10.2.

To further clarify the clustered regions and their spatial distribution characteristics, the results of the cluster and outlier analysis were visualized using ArcGIS 10.2 (Fig 3). The spatial distribution pattern of ω(Sn) is characterized by high-value clustering (HH), which is mainly concentrated in the southeastern and western regions of Yunnan Province. Among them, the southeastern part includes Jianshui County, Kaiyuan City, Gegiu City, Mengzi City and Maguan County, while the western part includes Tengchong City, Yingjiang County, Longling County, Lianghe County, Longchuan County and Mangshi City.

### Hotspot detection of ω(Sn) distribution

The analysis showed that the significance level of ω(Sn) is greater than 0.05, allowing the identification of positive and negative hotspots, representing counties with relatively high or low concentrations, respectively (Table 2). The number of counties with positive hotspot areas with ω(Sn) distribution is higher than the number of negative hotspot areas, of which there are 22 positive hotspot areas, accounting for 17%, and 15 negative hotspot areas, accounting for 11.6%.

**Table 2:** 2 Hot spot analysis result of ω(Sn).

ω(Sn)	Positive hot spot		Negative hot spot	
	count	%	count	%
	22	17%	15	11.6%

**Fig 4:**
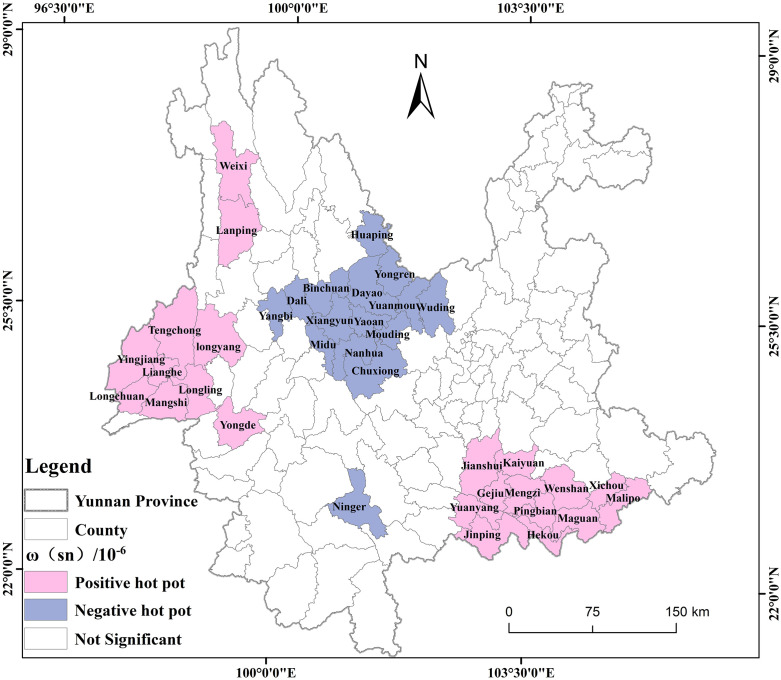
Spatial distribution of the ω(Sn) hotspot region in Yunnan Province. Created using ArcGIS 10.2.

To further visualize the spatial locations of the hotspot regions for ω(Sn), GIS was employed to generate spatial distribution maps (Fig 4). The positive hotspot areas of ω (Sn) distribution are mainly concentrated in the southeastern and northwestern regions of Yunnan Province. The southeastern part includes 12 counties, including Mengzi City, Kailuan City, Wanguo City, Wenshan City, Jianshui County, Yuanyang County, Jinping County, Pingbian County, Hekou County, Maguan County, Xichou County, and Ma’liipo County; the northwestern part includes 10 counties, including Mang City, Tengchong City, Lonyang District, Weixi County, Lamping County, Yingjiang County, Lianghe County, Longling County, Longchuan County, and Yongde County; and negative hotspot areas are mainly concentrated in the central region, including Dali City, Xiangyun County, Binchuan County, Maidu County, Yangbi County, Chuxiong City, Nanhua County, Muding County, Yaoan County, Dayao County, Wuding County, Yuanmou County, Huaping County, Yongren County, and 15 counties in Ning’er County.

### Spatial overlay analysis of ω(Sn) and longevity levels

**Fig 5:**
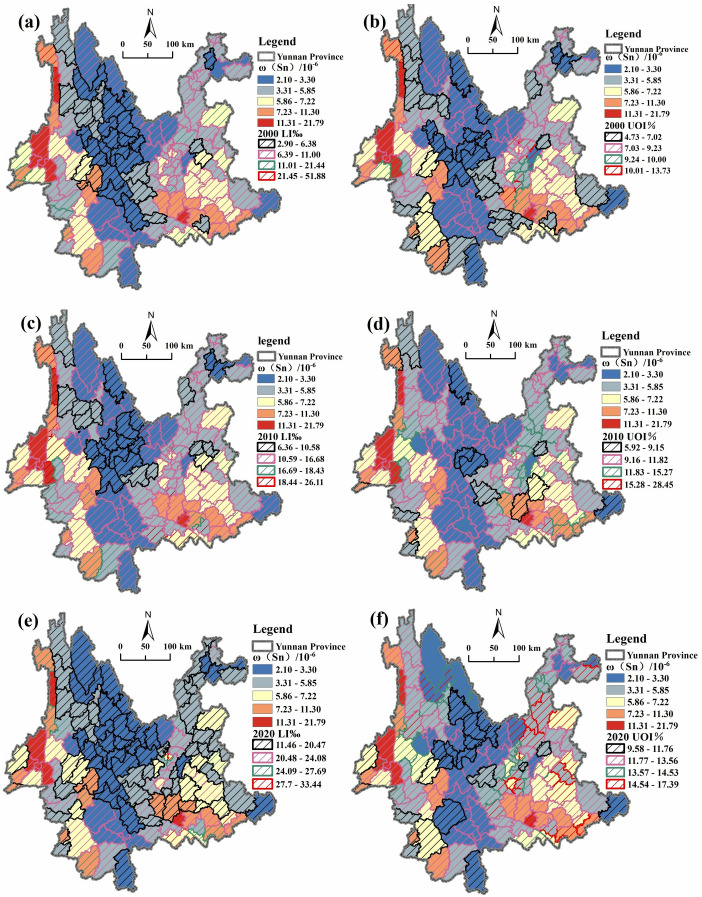
Overlay map of ω(Sn) and population longevity levels in Yunnan Province. Created using ArcGIS 10.2.

In order to better compare the longevity index and the Ultra-octogenarian Index of the counties in Yunnan Province with the national average, the average values of the longevity index (24.08, 16.68, and 11) and the Ultra-octogenarian Index (13.56, 11.82, and 9.23) measured for the fifth, sixth, and seventh times in the country were used as references. Then, according to the 15% above or below the average value in order, they were classified into 4 levels and a superimposed analysis graph of ω (Sn) with the population longevity level was generated (Fig 5). Most of the counties in Yunnan Province with LI‰ higher than the national level from 2000 to 2020 are in the eastern, western and southern regions, with ω(Sn) values in the range of 3.31–21.79 mg/kg; counties with LI‰ lower than the national level are mainly distributed in the central and northwestern regions, with a small portion randomly distributed, and their ω(Sn) values are in the range of 2.10–3.30 (Fig 5 a, c, e). Counties with UOI% higher than the national level in Yunnan Province from 2000 to 2020 are mostly in the eastern and western regions, except for a small random distribution, and their ω(Sn) values are within the interval of 3.31–21.79 mg/kg; counties with UOI% lower than the national level are mainly located in the central and northwestern regions, with a small random distribution, and their ω(Sn) values are within the interval of 2.10–3.30 (Fig 5 b, d, f).

In 2000, there were 17 counties in Yunnan Province with LI‰ higher than the national level, all of which were distributed in the interval of ω(Sn) > 3.3 mg/kg (Fig 5a); in 2010, there were 23 counties with LI‰ higher than the national level, and except for Yuanmou County (2.70 mg/kg), the remaining 22 were distributed in the interval of ω(Sn) > 3.3 mg/kg (Fig 5c); in 2020 There are 18 counties in Yunnan Province with LI‰ higher than the national level, all of which are distributed within the interval of ω(Sn) > 3.3 mg/kg (Fig 5e). In 2000, there were 24 counties in Yunnan Province with UOI% higher than the national level, all of which were distributed in the interval of ω(Sn) > 3.3 mg/kg (Fig 5b); in 2010, there were 39 counties with UOI% higher than the national level, of which 35 were distributed in the interval of ω(Sn) > 3.3 mg/kg. Although four counties were below the national level, namely, Yongping County (3.26 mg/kg), Binchuan County (3.00 mg/kg), Yuanmou County (2.70 mg/kg) and Ning’er County (2.16 mg/kg). Yongping and Binchuan counties were both close to the national level of 3.3 mg/kg (Fig 5d); there are 57 counties with UOI% higher than the national level in 2020, and 53 of them are distributed in the interval of ω(Sn) > 3.3 mg/kg.Four counties, namely, Yongping County (3.26 mg/kg), Shangri-La City (3.23 mg/kg), Weixin County (3.19 mg/kg), and Huaping County (3.19 mg/kg), though lower than the national level, are all close to the national level of 3.3 mg/kg (Fig 5f).

### Relationship between ω (Sn) and regional longevity levels

**Fig 6:**
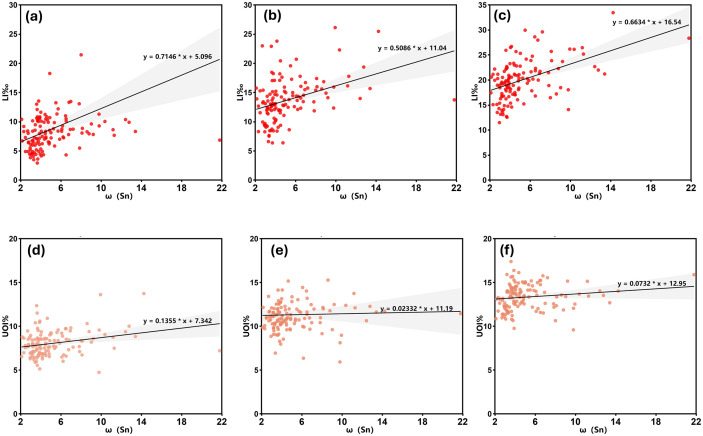
Scatter Plots of Correlations Between ω(Sn) and Population Longevity in Yunnan Province. **(2000: a, d; 2010: b, e; 2020: c, f).** Created using JetBrains PyCharm 2018.3.7.

To more intuitively illustrate the relationship between ω(Sn) and longevity levels, a bivariate scatter plot was created using JetBrains PyCharm programming software, and a linear fit was performed (Fig 6). There was a positive correlation between ω(Sn) and LI‰ in Yunnan Province from 2000 to 2020. As ω(Sn) increases, LI‰ generally showed an upward trend. This suggests that ω(Sn) may play a positive role in improving regional longevity levels, particularly evident in Fig a, where the slope is the steepest and the regression band is the widest, indicating a strong correlation and some variability(Fig 6 a, b, c); although ω(Sn) and UOI% both exhibit a positive correlation trend, their slopes are only 0.1355, 0.02332, and 0.0732, respectively, indicating that the influence of ω(Sn) on regional longevity levels is relatively weak. The correlation between ω(Sn) and UOI% is less significant than that with LI‰. The narrower regression band of UOI% further indicates that the data fluctuations are smaller and the trend is more stable(Fig 6 d, e, f).

Scatter plots are only used to show the relationship trends between sample variables, but they cannot directly determine the correlation between sample variables at the regional statistical level. Therefore, it is necessary to conduct significance tests on sample variables to verify their correlation. To ensure the applicability of the statistical methods and the accuracy of the results, the Kolmogorov-Smirnov test was used to test the normality of all data. The test results showed that some of the data did not meet the assumption of normal distribution (P < 0.05). In view of this, the present study used Spearman’s rank correlation coefficient for correlation analysis to more accurately assess the relationship between ω(Sn) and longevity levels (Table 3).

**Table 3:** Correlation analysis between ω(Sn) and longevity levels.

Element	Year	Indicators	r	P level	n
ω(Sn)	2000	LI%	0.410^**^	<0.001	129
		UOI%	O.248^**^	<0.001	
	2010	LI%	0.446^**^	<0.001	
		UOI%	0.149^*^	0.046	
	2020	LI%	0.472^**^	<0.001	
		UOI%	0.246^**^	<0.001	

**Indicates significance at the 0.01 confidence level; *indicates significance at the 0.05 confidence level.

As shown in Table 3, from 2000 to 2020, there was a significant positive correlation between ω (Sn) and LI‰ in Yunnan Province (P < 0.01), with correlation coefficients (r) of 0.410, 0.446, and 0.472, respectively, and the correlation continued to strengthen. Similarly, from 2000 to 2020, ω (Sn) showed a significant positive correlation with UOI%, with correlation coefficients of 0.248 (P < 0.01), 0.149 (p < 0.05), and 0.246 (P < 0.01).

Spearman’s rank correlation preliminarily reveals a positive correlation between ω(Sn) and LI‰ and UOI%. On this basis, in order to further verify the specific degree of influence of this relationship, GraphPad Prism 10.4 was utilized to construct a one-way linear regression model with LI‰ and UOI% as dependent variables and ω(Sn) as independent variable, respectively.

As shown in Table 4, during the period of 2000, ω (Sn) had a significant positive effect on LI‰ (P < 0.01), with a regression coefficient (B) of 0.715, and the model could explain approximately 12.4% of the variance in the dependent variable. Further analysis of the 2010 data shows that the regression coefficient of ω (Sn) decreased to 0.509 but remained statistically significant (P < 0.01), with the model’s explanatory power for the dependent variable increasing to 15.2%. By 2020, the regression coefficient of ω(Sn) increased to 0.664, with the same level of significance of P < 0.01, indicating that its positive impact on LI‰ further strengthened during this period, and the model’s explanatory power for the dependent variable improved to 22.8%.

**Table 4:** Linear regression analysis of ω (Sn) on LI‰.

Year	LI‰	B	Standard error	t value	P level	VIF	R^2^
2000	ω(Sn)	5.096	0.971	5.247	<0.001	1.000	0.131
		0.715	0.164	4.368	<0.001		
2010	ω(Sn)	11.044	0.632	17.464	<0.001	1.000	0.152
		0.509	0.107	4.775	<0.001		
2020	ω(Sn)	16.539	0.644	25.686	<0.001	1.000	0.228
		0.664	0.108	6.117	<0.001		

As shown in Table 5, during the year 2000, ω(Sn) had a significant positive effect on UOI% (P < 0.01), with a regression coefficient (B) of 0.136, and this model explained approximately 7% of the variance in the dependent variable. However, in 2010 and 2020, the effect of ω(Sn) on UOI% was not significant, with regression coefficients of 0.023 and 0.073, respectively. Additionally, the R² values for these two time periods were 0.001 and 0.021, respectively, indicating that the explanatory power of ω(Sn) for the dependent variable was very limited.

**Table 5:** Linear regression analysis of ω (Sn) on UOI%.

Year	UOI%	B	Standard error	t value	P level	VIF	R^2^
2000	ω(Sn)	7.342	0.261	28.173	<0.001	1.000	0.070
		0.136	0.044	3.087	<0.001		
2010	ω(Sn)	11.190	0.475	23.562	<0.001	1.000	0.001
		0.023	0.080	0.026	0.771		
2020	ω(Sn)	12.954	0.265	48.843	<0.001	1.000	0.021
		0.073	0.045	1.639	0.104		

Although no statistically significant associations were demonstrated between ω(Sn) and UOI% in 2010 and 2020 under the current study design and data, the correlations between ω(Sn) and LI‰ all passed the significance test at the confidence level of 0.01, i.e., a non-negligible association between ω(Sn) and regional population longevity levels was revealed at the regional statistical level.

## Discussion

In a region such as Yunnan Province, with diverse geologic landforms, ecology and soil types, and significant differences between the degree of resource development and the level of economic development and medical care, the reasons for exhibiting a statistically significant positive correlation statistical pattern between ω(Sn) and the regional longevity level are worthy of further investigation.

**Fig 7:**
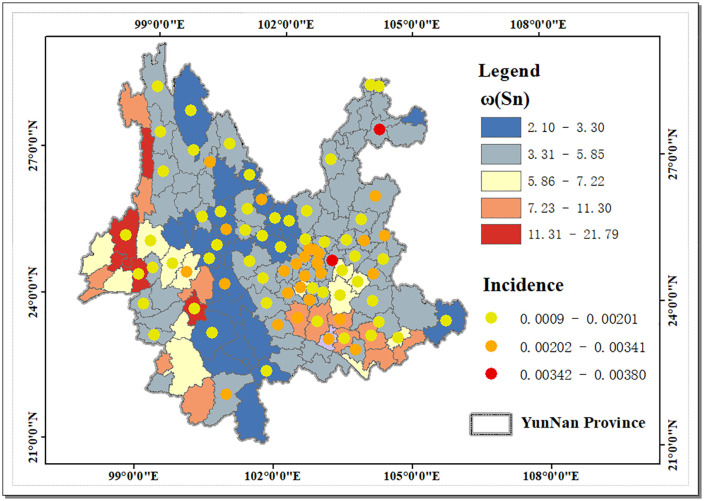
Distribution of ω(Sn) and cancer incidence in Yunnan Province. Created using ArcGIS 10.2.

Relevant reports indicate that cancer has become one of the most lethal conditions at present [45]. Existing studies have shown that Sn levels in the environment or tissues may be significantly correlated with cancer incidence. Several ex vivo and in vivo experiments have confirmed that Sn has biological activities to inhibit cancer cell proliferation, induce apoptosis, and block metastasis [26]. Clinical samples also showed that in many chronic degenerative diseases, such as breast cancer, lung tumors, colorectal cancer and other patients, the Sn content in tumor tissues was significantly lower than that in other normal tissues [46]. Is there also a relationship between ω(Sn) and cancer incidence at the county scale in Yunnan Province? The article selected the data of new cancer cases in Yunnan Province from the Annual Report of China Cancer Registry 2021 [47] to calculate the cancer incidence rate (per 100,000 people), and used the global average of cancer incidence rate (0.00201) as a reference [48], and the results of the analysis are shown in Fig 7. It can be seen that there are 29 counties in Yunnan Province with cancer incidence rates higher than the global average, except for 7 counties, namely, Wuhua, Panlong, Hongta, Fengqing, Gejiu, Kaiyuan, and Shiping, and the remaining 22 counties are distributed in the range of ω(Sn) values <5.85 mg/kg, accounting for 75%; while the counties with cancer incidence rates lower than the global level generally have ω(Sn) values >5.85 mg/kg, which to some extent reflects that higher ω(Sn) may have an inhibitory effect on the cancer risk of local residents, thus affecting the regional longevity level.

At the county scale, although regions with high ω(Sn) showed a clear spatial association with higher longevity levels, and most of the counties were negatively correlated with cancer incidence, some of the positive hotspot regions did not exactly correspond to the distribution of longevity levels and cancer incidence. This phenomenon suggests that the relationship between ω(Sn) and longevity level may not be a simple linear relationship, but is influenced by a combination of factors. It is worth noting that most of Yunnan Province has a beautiful environment, favorable climate, and abundant flora and fauna, which may have a positive impact on the longevity of the population in the region [49,50]. However, the special natural environments in some regions, such as high altitude, thin air and low oxygen content in the northwest, predispose to cardiovascular and respiratory diseases [51–53], which can reduce the level of longevity. In addition, natural disasters, traffic accidents, as well as socio-economic conditions and medical care levels in each region are also important factors that affect the health and longevity levels of the population. Meanwhile, there are large differences in the natural environment and the level of social development in 129 counties within Yunnan Province, including climate [1,54], air quality [55], drinking water quality [56,57], soil quality [17,10], and the degree of development, etc., which may affect the migration and transformation of Sn in the environment, as well as the strength of interaction with other elements, and thus affect the environmental Sn exposure levels.

The above analyses indicate that ω(Sn) is significantly and positively associated with longevity levels at the county scale, and may have a suppressive effect on the risk of cancer in regional populations. These findings provide strong regional evidence for understanding the relationship between regional longevity levels and environmental factors, and may provide an important basis and reference for the selection of sites for health and wellness tourism destination, as well as for improving the health status of the regional populations and their longevity levels, and for regional health policymaking. However, the generalizability of these results may be affected by the unique geographic environment and demographic characteristics of the study region. When applying the results of the study to other regions, it is necessary to validate and analyze them in the context of the actual local situation, and to consider the reasonableness and prudence of logical reasoning. Moreover, there are some limitations in this study. On the one hand, the time span of the research statistics is long, and it mainly focuses on ω(Sn) at the county scale in Yunnan Province without fully considering the dynamic changes of this factor over a longer period of time, which may slightly affect the precision of the results. On the other hand, further research is needed to investigate whether ω(Sn) has a synergistic effect with other factors on population longevity, or whether other factors influence the level of Sn exposure and thus have an impact on population longevity. These complex relationships deserve to be explored and studied in depth in the future.

## Conclusions

Based on the geochemical survey data of Yunnan Province and the fifth, sixth and seventh population census data of China, the article utilizes ArcGIS spatial analysis and mathematical statistics to analyze the spatial differentiation characteristics of ω(Sn) on the regional scale of Yunnan Province, and focuses on the relationship between ω(Sn) and the regional longevity level, with the main conclusions as follows:

(1) There is a close correlation between ω(Sn) and regional longevity levels, i.e., regions with high ω(Sn) have both LI‰ and UOI% at higher levels within Yunnan Province. This may also imply that ω(Sn) influences regional population health to some extent.
(2) Spearman’s rank correlation shows that ω(Sn) has a significant positive correlation with LI‰, and this correlation is increasing; in addition, ω(Sn) also has a significant positive correlation with UOI%. Linear regression analysis shows that ω(Sn) has a significant positive effect on LI‰, but has a significant positive effect on UOI% only in 2000, and the effect is not significant from 2010 to 2020.
(3) At the county scale, ω(Sn) shows a close correlation with cancer incidence, and it is tentatively hypothesized that higher ω(Sn) may have an inhibitory effect on the incidence of certain cancers and thus have an impact on regional longevity levels.

Although this study revealed the relationship between ω (Sn) and longevity levels of the population at the county scale as well as cancer incidence, a clear causal relationship has not yet been established. Future studies need to synthesize knowledge from multiple disciplines and fields to investigate in depth the specific mechanisms of Sn action in the natural environment in the human body and the interactions between Sn and other trace elements. At the same time, differences between different populations and environments should be taken into account to accurately assess the impact of Sn on human health longevity. The ultimate goal is to control and reduce the occurrence of diseases, improve human longevity, and even promote the development of Sn-related health and tourism industries. This work will be challenging, but far-reaching.
